# It takes two

**DOI:** 10.7554/eLife.11399

**Published:** 2015-10-06

**Authors:** Teresa Nicolson

**Affiliations:** Oregon Hearing Research Center and the Vollum Institute, Oregon Health and Science University, Portland, United Statesnicolson@ohsu.edu

**Keywords:** deafness, stereocilia, myosin, hearing, actin, inner ear, mouse

## Abstract

Two forms of an unconventional myosin motor protein have separate functions in the growth and maintenance of hair bundles in auditory hair cells.

**Related research article** Fang Q, Indzhykulian AA, Mustapha M, Riordan GP, Dolan DF , Friedman TB, Belyantseva IA, Frolenkov GI, Camper SA, Bird JE. 2015. The 133-kDa N-terminal domain enables myosin 15 to maintain mechanotransducing stereocilia and is essential for hearing. *eLife* **4**:e08627*.* doi: 10.7554/eLife.08627

**Image** The large isoform of myosin 15 (green) localizes predominately at the tips of short stereocilia (magenta), but not tall stererocilia, in inner hair cells in the cochlea of mice

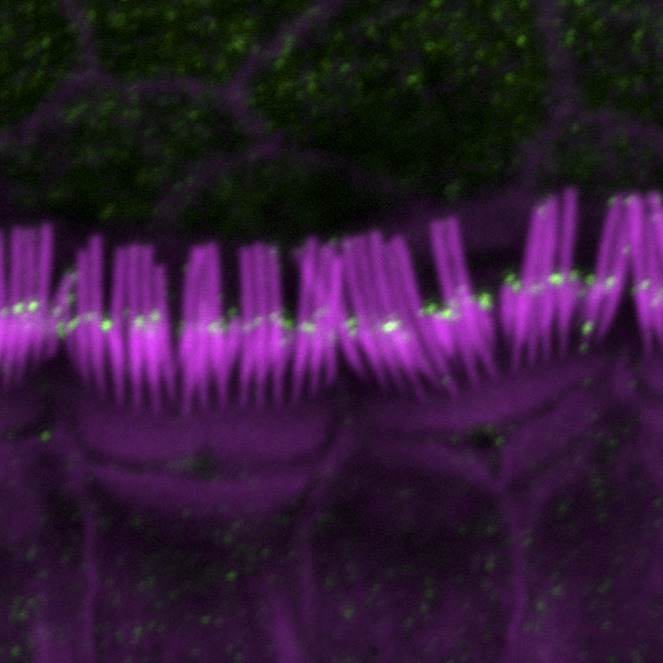

A major challenge in hearing research is to understand how structures known as ‘hair bundles’ are formed in the cochlea. Hair bundles have a crucial role in the detection of sound and the conversion of mechanical signals (that is, sound waves) into electrical signals. The cochlea contains two types of hair cells – inner and outer – and a hair bundle protrudes from the top of every hair cell. Each hair bundle consists of a collection of smaller hair-like structures called stereocilia that line up in rows within the bundle to form a structure that resembles a staircase (Figure 1). The stereocilia are filled with filaments made of the protein actin.

**Figure 1.**
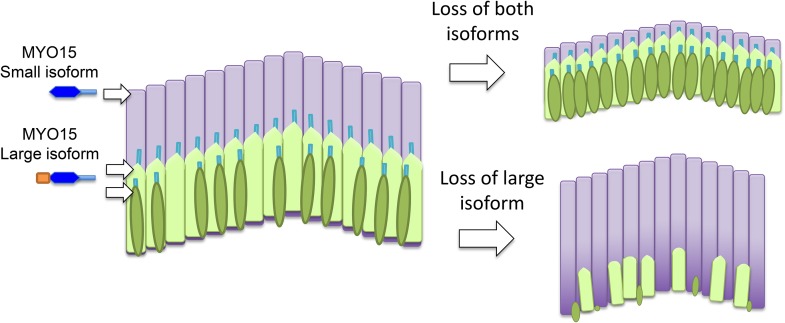
The roles of the two isoforms of myosin 15 (MYO15) in hair bundles. Left: Schematic depiction showing the three rows of stereocilia in a normal hair bundle, with the first row (dark green) being the shortest and the third row (pale purple) being the tallest. This difference in height results in a characteristic staircase-like structure. The stereocilia in the first two rows mediate the process of mechanotransduction, and the large isoform of myosin 15 localizes to the tips of these stereocilia; the small isoform is found primarily in the taller stereocilia in the third row. Right: When both isoforms are defective or absent, the stereocilia in the third row do not reach their normal height (top). If the N-terminal extension in the large isoform is absent in mice, hair bundles form normally but some of the stereocilia in the first two rows degenerate in older animals (bottom). The large isoform of myosin 15 has a large extension (shown in orange) at its N-terminus.

Through studies of deaf patients, geneticists have made remarkable progress in identifying genes that are required for hearing (see hereditaryhearingloss.org). Many of the corresponding proteins are important for the function of hair cells and more than a dozen of them have roles in the hair bundle; these proteins include several myosin motor proteins that differ from the conventional myosin motors that are found in muscle cells. Hair cells actually produce two versions (or isoforms) of one of these unconventional myosin motors, myosin 15 (Wang et al., 1998; Liang et al., 1999). One of these isoforms has a large (134kD) extension at its N-terminus, but the role played by this extension in hair cells has long been a mystery.

A clue to the importance of the extension is provided by the fact that mutations in the gene (exon 2) that encodes the additional amino acids in the extension cause deafness in humans (Nal et al., 2007). To explore the role of this extension Jonathan Bird and co-workers – including Qing Fang as first author – have compared mice in which the myosin 15 proteins have the extension (isoform 1) and mice in which they do not (isoform 2; Fang et al., 2015).

Previously our knowledge about the function of myosin 15 was based on studies of mice with a mutant *shaker2* gene: this mutation leads to defective hair cells in both the cochlea and the vestibular system, which is the part of the ear that controls balance. (The name *shaker* was coined to describe the unsteady movements seen in these mice). The *shaker2* mutation effects both isoforms of myosin 15 and prevents the stereocilia growing beyond a certain height (Probst et al., 1998). The staircase-like structure seen in normal hair bundles is not seen in the *shaker2* mice.

Experiments with an antibody that recognizes both isoforms suggest that myosin 15 is located at the tips of the stereocilia (Belyantseva et al., 2003). The *shaker2* phenotype suggests that myosin 15 promotes the growth of stereocilia, presumably by working as an actual motor that interacts with actin filaments (Bird et al., 2014). However, the details of how this happens are not fully understood, although it might depend on proteins that are transported to the growing tip by myosin 15 (Belyantseva et al., 2005; Zampini et al., 2011).

To examine the role played by the large extension in isoform 1, Fang, Bird and colleagues – who are based at the University of Michigan, the National Institute on Deafness and Other Communication Disorders, and the University of Kentucky – generated an antibody that is specific to this isoform and used it to investigate the effects of deleting the exon 2 gene (Fang et al., 2015). Surprisingly, they found that isoform 1 is restricted to the first two rows of stereocilia in inner hair cells (Figure 1). In outer hair cells, on the other hand, isoform 1 is also found at the tall stereocilia in the third row. As for isoform 2, it is mainly present in the third row in inner hair cells.

Finding the two isoforms in different locations came as a surprise, but it could help to explain why deletion of the N-terminus and *shaker2* mutations lead to different phenotypes. *Shaker2* mutations affect both isoforms and lead to short hair bundles. Deletion of the N-terminus does not affect the length of stereocilia: rather, the hair bundles develop normally at first, but the first two rows of stereocilia then wither away. This suggests that the large isoform is important for the maintenance of a subset of the stereocilia: in particular, it maintains the stereocilia are involved in converting sound energy into an electrical signal in the inner part of the cochlea.

This conversion process, which is called mechanotransduction, is largely present in both the *shaker2* mutants and in the mice in which the N-terminus has been deleted, albeit with some subtle differences. This phenotype suggests that myosin 15 is not directly involved in mechanotransduction: however, it seems that the large isoform of myosin 15 can recognize and accumulate at sites where this process takes place. The localization pattern of myosin 15 observed in the outer hair cells reinforces the idea that some form of membrane tension is required for accumulation of the large isoform.

A similar result was found with another protein (called sans) that is required for growth of stereocilia: deleting sans after hair bundles had fully formed caused the first two rows of stereocilia to shrink over time (Caberlotto et al., 2011). Sans interacts with the mechanotransduction machinery in hair cells (Lefèvre et al., 2008), and the loss of sans has a more dramatic effect on mechanotransduction than the loss of myosin 15. Nevertheless, these two cases suggest that it is possible to uncouple the different roles of various proteins in development and in the subsequent maintenance of mechanically-sensitive stereocilia in hair bundles. It will be interesting to see whether other short bundle mutants may have a similar phenotype, if given the chance.
